# Quantitative assessment of airway wall thickness in COPD patients with interstitial lung abnormalities

**DOI:** 10.3389/fmed.2023.1280651

**Published:** 2023-12-07

**Authors:** Yingying Ji, Leqing Chen, Jinrong Yang, Xiangying Yang, Fan Yang

**Affiliations:** ^1^Department of Radiology, Union Hospital, Tongji Medical College, Huazhong University of Science and Technology, Wuhan, Hubei, China; ^2^Hubei Province Key Laboratory of Molecular Imaging, Wuhan, China

**Keywords:** interstitial lung abnormalities, idiopathic pulmonary fibrosis, COPD, quantitative CT, lung function

## Abstract

**Background:**

Whether the airway is involved in the pathogenesis of interstitial lung abnormalities (ILA) is not well understood. Also the impact of ILA on lung function in COPD patients remains controversial. We aimed to assess the quantitative CT measurements of airway wall thickness (AWT) and lung function according to ILA status in COPD patients.

**Methods:**

157 COPD patients discharged from our hospital from August 1, 2019 through August 31, 2022 who underwent chest CT imagings and pulmonary function tests were retrospectively enrolled. Linear regression analysis and multiple models were used to analyze associations between quantitative assessment of airway wall changes and the presence of ILA.

**Results:**

In 157 COPD patients, 23 patients (14.6%) had equivocal ILA, 42 patients (26.8%) had definite ILA. The definite ILA group had the highest measurements of Pi10 (square root of theoretical airway wall area with a lumen perimeter of 10 mm), segmental AWT and segmental WA% (percentage of wall area), whereas the no ILA group had the lowest measurements of Pi10, segmental AWT and segmental WA%. In the adjusted analyses (adjusted by age, sex, body mass index, smoking intensity, COPD GOLD stage, lung function, slice thickness and scanner type), compared to COPD patients without ILA, the measurements of Pi10, segmental AWT and segmental WA% were higher in definite ILA group with differences of 0.225 mm (*p* = 0.012), 0.152 mm (*p* < 0.001), 4.8% (*p* < 0.001) respectively. COPD patients with definite ILA tended to have higher FEV1% predicted, FVC% predicted and lower MMEF_75/25_% predicted, but there were no statistically differences among the three groups.

**Conclusion:**

Our study demonstrates the higher AWT measures in COPD patients with ILA compared to the patients without ILA. These findings suggest that the airway may be involved in the pathogenesis of ILA.

## 1 Introduction

Interstitial lung abnormalities (ILA) are asymptomatic radiological abnormalities detected by chest CT, shown as high-attenuation areas in the lung field. ILA are usually associated with interstitial lung disease (ILD) and idiopathic pulmonary fibrosis (IPF), and can be considered as the subclinical phase of ILD (1, 2). It is becoming increasingly clear that certain forms of ILD progress from an asymptomatic or “subclinical” stage to a final clinical diagnosis. IPF is a progressive, destructive disease with unknown causes and few treatment options, and its incidence has gradually increased in recent years (3). The molecular mechanisms of ILD and IPF have garnered increased attention due to the historical lack of understanding of their etiology and natural history, as well as the absence of effective treatments (4). Previous studies have shown that the undiagnosed patients with ILA share several similar characteristics with clinically significant ILD patients, including reduced lung volume, limited function, increased lung symptoms, histopathological changes, and molecular characteristics, as well as similar but milder syndromes and genetic and genomic similarities with IPF patients, and ILA may have a common pathogenesis with ILD and IPF (5). Therefore, a full understanding of ILA can improve the understanding of the natural process of ILD and IPF, and make it possible for the early management and timely intervention of these diseases.

Several studies have shown that IPF may be caused by abnormal behavior of alveolar epithelial cells combined with excessive fibroblast activation (3, 6). It has been gradually observed that bronchiolar epithelial cells and broncho-alveolar junctions are related targets of cell damage in IPF, thus the correlation between airway epithelial biology and IPF has been gradually realized (4). Therefore, this study hypothesized that the airway might be involved in the pathogenesis of ILA and tested this hypothesis by measuring airway wall thickness (AWT) in ILA patients using quantitative CT.

To our knowledge, studies using quantitative CT measurements of AWT have focused on COPD, while few studies on ILA (7, 8). And because ILA are relatively common in COPD patients, we also want to investigate whether the prevalence of ILA exacerbates the lung function in COPD patients, as some studies have demonstrated (2, 9). Thus, we hypothesized that the COPD patients with equivocal ILA and definite ILA had higher AWT and lower lung function compared to those without ILA. We assessed the quantitative CT measurements of AWT and lung function in COPD patients to evaluate the hypothesis.

## 2 Materials and methods

### 2.1 Study populations

**Figure 1:**
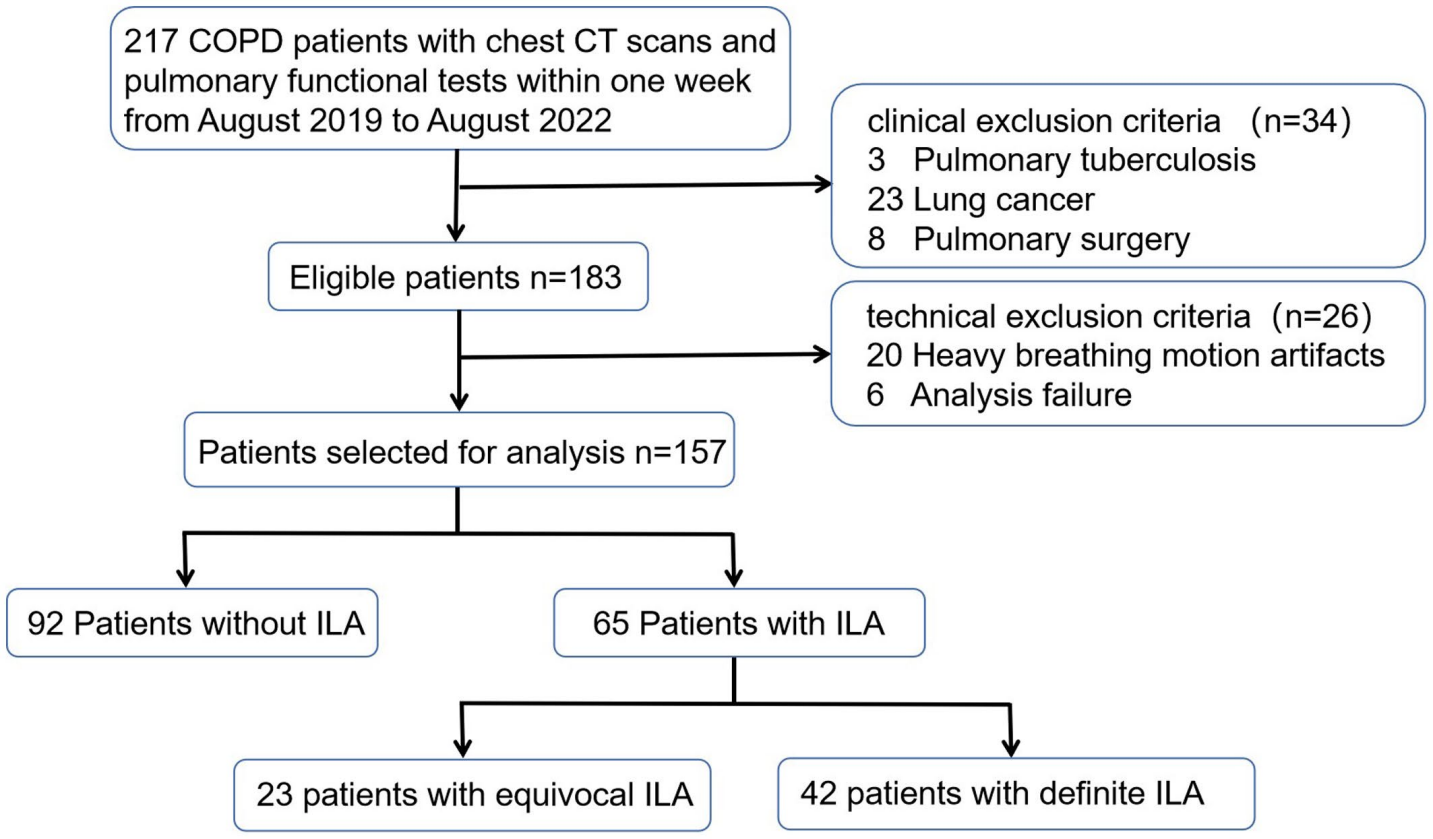
Flow diagram of the study population. ILA, interestitial lung abnormality.

The Medical Ethics Committee of our hospital approved this study (NO.0271–01), and formal consent was not required as this was a retrospective study. Patients with COPD discharged from our hospital from August 1, 2019 through August 31, 2022 were retrospectively enrolled. The inclusion criteria for this study were as follows: (1) COPD patients diagnosed with spirometric criteria (post-bronchodilator FEV1/FVC < 0.7), (2) patients who had completed chest CT scans and pulmonary functional tests (PFTs) within one week. The exclusion criteria included: (1) other chronic respiratory diseases such as asthma, pulmonary tuberculosis, or lung cancer, (2) a history of pulmonary surgery or thoracic deformity, (3) infectious diseases affecting radiological changes in the lung parenchyma. A total of 157 patients were ultimately enrolled. The detailed flow chart was shown as Figure 1.

### 2.2 CT scanning

All participants underwent imagings with IQon Spectral CT (Philips Healthcare, Best, The Netherlands) or SOMATOM Definition AS+ (Siemens Healthineers. Forchheim, Germany) at full inspiration in the supine position. The instrument settings were as follows: detector collimation width of 64 × 0.6 mm or 128 × 0.6 mm; tube voltage of 120 kV; tube current of 40–80 mA, the tube current was regulated by automatic exposure control system (CARE Dose 4D; Siemens Healthineers) or (DoseRight, Philips Healthcare); slice thicknesses of 1.25 mm or 2.00 mm and internals of 1.25 mm or 2.00 mm, slice thicknesses of 2.00 mm accounts for 7/92 (7.6%) in the no ILA group, 2/23 (8.7%) in the equivocal ILA group, 3/42 (7.1%) in the definite ILA group; All images were reconstructed with lung and soft tissue kernels for quantitative analysis, reconstruction kernel of B70f kernel and a mediastinal B30f kernel.

### 2.3 Pulmonary functional tests

All PFTs were performed using Master Lab equipment (CareFusion, Hoechberg, Germany), Spirometry for FEV1 and FVC was performed before and 15 min after inhalation of 200 mg salbutamol following the American Thoracic Society Committee guidelines (10). The following values were recorded: FEV1/FVC, FEV1, FVC and maximum mid-expiratory flow (MMEF75/25). All values were expressed as a percentage of the predicted value. All PFTs and CT scans were completed within one week.

### 2.4 Imaging review

Three radiologists with 5, 7, and 15 years of experience visually assessed chest CT scans for ILA, blinded to any patient-specific information. Findings definite for ILA were defined as nondependent changes that affected more than 5% of any lung zone, including nondependent ground-glass opacity (GGO) or reticular abnormality, diffuse centrilobular nodularity, honeycombing, traction bronchiectasis, nonemphysematous cysts, or architectural distortion. Focal or unilateral ground-glass attenuation, focal or unilateral reticulation, and patchy ground-glass abnormalities that affected less than 5% of the lung were considered equivocal findings (2). Discrepancies in CT diagnosis or ILA characterization were resolved by consensus.

In addition, the extent of ILA were graded for every lobe using the following five-point scale: score of 0, absent; score of 1, 1 to 25% involvement; score of 2, 26 to 50% involvement; score of 3, 51 to 75% involvement; and score of 4, 76 to 100% involvement. Total ILA score ranges from 0 to 24 with the lingular segment counted separately (2).

### 2.5 Quantitative CT measurements

**Figure 2:**
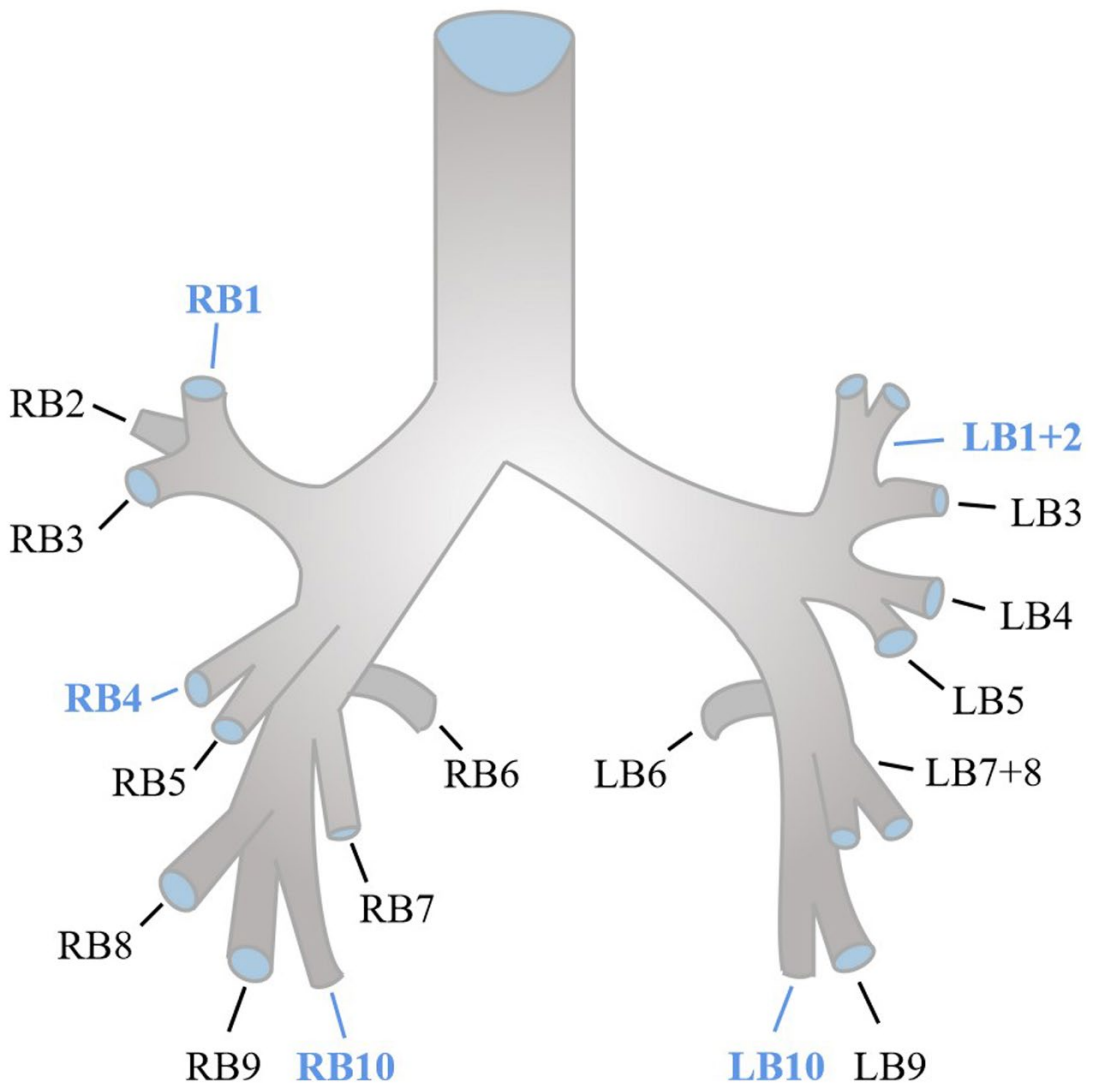
Anatomy of segmental bronchi of normal segmented airway tree.

Quantitative CT measurements were evaluated using the COPD Analysis package (Philips IntelliSpace Portal). The extent of emphysema was quantified by evaluating the areas of the lung with attenuation less than −950 Hounsfield units (HU) at full inspiration, commonly referred to as LAA-950 (10). We used an integral-based method for airway measurements (11). To calculate the segmental airway measurements, the average of five segmental airways (RB1, RB4, RB10, LB1+2, and LB10) along the middle third of each airway was taken (10, 12), the anatomy of normal segmental bronchi was shown as Figure 2. The segmental wall area percentage (WA%) was determined by taking the average of the five segmental airways and calculating (WA/total cross-sectional area) × 100. Pi10, which was assessed from the regression line calculated by plotting the square root of the wall area and the lumen perimeter of the airway, was defined as the square root of the wall area of a theoretical airway with a lumen perimeter of 10 mm (13).

### 2.6 Statistical analysis

The study reported continuous variables as mean ± SD and categorical data as percentages. Differences in continuous variables between groups were tested using Kruskal-Wallis or ANOVA tests depending on the normality of distribution, and the LSD-t method was used for post-hoc comparisons. Chi-square tests were used to compare differences in categorical data between groups. Linear regression analysis was conducted to evaluate the associations between the quantitative assessment of airway changes (Pi10, segmental AWT, segmental WA%) and the presence of ILA. Multivariate models were used to adjust for potential confounding factors such as age, sex, BMI, smoking intensity, classification of Chronic Obstructive Pulmonary Disease (GOLD), lung function (FEV1/FVC, FEV1 percent predicted, FVC percent predicted, MMEF75/25 percent predicted), slice thickness and scanner type. Logistic regression analysis was conducted to evaluate the associations between age and the presence of ILA, potential confounding factors including sex, BMI, smoking intensity, COPD GOLD Stage were used for multivariate models. Statistical analysis was performed using SPSS software (version 27.0), and the significance level was set at *p* < 0.05 (two-tailed).

## 3 Results

### 3.1 Baseline characteristics

After assessing 217 COPD patients, 157 eligible patients completed both chest CT scanning and PTFs within one week and were included in the study (Figure 1). Table 1 presents the baseline characteristics of the study population. Of these COPD patients, 92 (58.6%) patients showed no evidence of ILA, 23 (14.6%) patients showed equivocal ILA, and 42 (26.8%) patients had definite ILA. Most of study populations were men (90%) with the mean age of 63.2 years. Similar to previous studies (2, 7), the COPD patients with ILA tended to be older, and increased age (OR adjusted = 1.069, 95%CI = 1.024–1.117, *p* = 0.003) was associated with definite ILA (Supplementary Table S1). For lung function, higher FEV1% predicted, FVC% predicted and lower MMEF_75/25_% predicted were noticed in the definite ILA patients, but all of the included lung function and the smoking intensity showed no statistically differences between these groups.

**Table 1:** Comparison of baseline demographics, lung function, quantitative CT measurements by ILA.

	No ILA (*n* = 92)	Equivocal ILA (*n* = 23)	Definite ILA (*n* = 42)	P0	P1	P2	P3
Age (year)	61.5 ± 9.0	64.0 ± 7.1	66.5 ± 8.8	0.008	0.610	0.837	0.007
Male sex (%)	85 (92.4)	21 (91.3)	35 (83.3)	0.266	0.572	0.312	0.102
BMI (kg/m^2^)	23.0 ± 3.2	23.5 ± 3.4	23.3 ± 3.0	0.721	1.000	1.000	1.000
Smoking intensity (pack-y)	16.1 ± 21.6	15.0 ± 23.5	15.3 ± 19.1	0.962	1.000	1.000	1.000
Baseline lung function	…	…	…	…	…	…	…
FEV1/FVC	61.2 ± 10.4	62.8 ± 8.6	61.9 ± 8.6	0.764	1.000	1.000	1.000
FEV1 (%_pred_)	84.1 ± 21.9	86.0 ± 18.2	86.0 ± 23.8	0.877	1.000	1.000	1.000
FVC (%_pred_)	107.7 ± 19.1	107.6 ± 15.3	108.9 ± 20.6	0.940	1.000	1.000	1.000
MMEF_75/25_ (%_pred_)	35.0 ± 15.3	33.9 ± 14.5	33.8 ± 12.7	0.903	1.000	1.000	1.000
Imaging	…	…	…	…	…	…	…
LAA_−950_ (%)	…	…	…	…	…	…	…
Whole lung (%)	5.94 ± 7.13	3.60 ± 4.95	4.85 ± 6.01	0.276	0.389	1.000	1.000
Upper lobes (%)	7.91 ± 10.43	3.28 ± 3.98	7.06 ± 9.59	0.118	0.003	0.087	0.954
Lower lobes (%)	4.88 ± 6.3	3.73 ± 6.21	3.46 ± 4.4	0.371	0.397	0.862	0.194
Pi10(mm)	5.15 ± 0.37	5.27 ± 0.57	5.39 ± 0.55	0.014	0.714	0.768	0.030
Segmental AWT (mm)	1.20 ± 0.16	1.31 ± 0.20	1.36 ± 0.20	<0.001	0.031	0.778	<0.001
Segmental LD (mm)	5.04 ± 0.71	5.05 ± 0.77	4.86 ± 0.89	0.419	1.000	0.988	0.633
Segmental LA (mm^2^)	21.21 ± 5.91	21.39 ± 6.18	20.03 ± 7.21	0.563	0.903	0.408	0.317
Segmental WA%	54.37 ± 6.18	56.62 ± 6.38	59.19 ± 7.09	<0.001	0.411	0.382	<0.001
TLCCT (L)	5.50 ± 1.08	4.90 ± 0.96	4.64 ± 1.23	<0.001	0.061	1.000	<0.001

Data are presented as No. (%) or mean ± SD unless otherwise indicated. BMI, Body mass index; FEV1, forced expiratory volume in 1 s; FVC, forced vital capacity; MMEF_75/25_, maximum mid-expiratory flow between 25–75%; LAA_−950_, Percentage area with CT attenuation values less than − 950 HU at inspiration; Pi10, square root of the wall area of a theoretical airway with internal perimeter of 10 mm; AWT, airway wall thickness; LD, lumen diameter; LA, lumen area; WA%, wall area percent; TLCCT, total lung capacity on CT. Segmental AWT, segmental LD and segmental WA% were calculated as the average of five segmental airway (RB1, RB4, RB10, LB1+2,and LB10). Lingula lobe was excluded from the calculation of the upper lobes. P0 is the *p* value of the three groups. P1 is the *p* value of no ILA group and the equivocal ILA group. P2 is the *p* value of the equivocal ILA group and definite ILA group. P3 is the *p* value of no ILA group and the definite ILA group.

**Figure 3:**
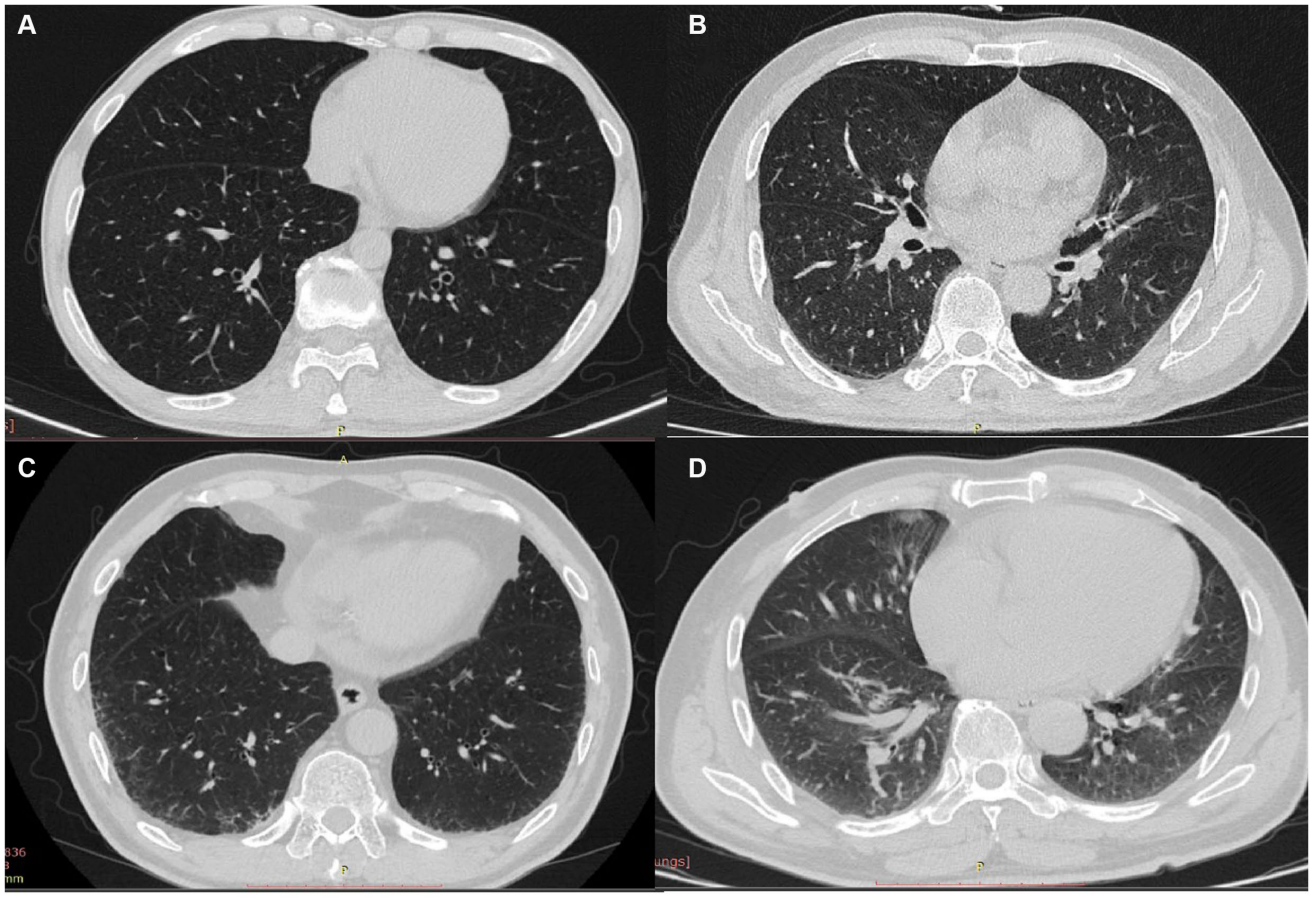
**(A–D)** CT findings of COPD patients without ILA and with equivocal/definite ILA; patients without ILA **(A)** and characteristic images of equivocal/definite ILA **(B–D)**, including unilateral reticulation **(B)**, bilateral reticular opacities **(C)** and nondependent group-glass opacity **(D)**.

Representative imaging findings of COPD patients without ILA and with equivocal/definite ILA are shown in Figure 3. Table 2 presents radiologic findings of chest CT scans in patients with equivocal and definite ILA. The most common radiologic findings of equivocal and definite ILA patients were focal or unilateral reticulation (73.9%) and nondependent ground-glass opacity (59.5%), respectively. The ILA scores of definite ILA tended to be higher than the patients with equivocal ILA (1.22 ± 0.52 vs. 2.93 ± 1.72).

**Table 2:** Radiologic findings in patients with equivocal and definite ILA.

Radiologic finding	Equivocal ILA (*n* = 23)	Definite ILA (*n* = 42)
Equivocal ILA		
Focal or unilateral GGO	5 (21.7)	1 (2.4)
Focal or unilateral reticulation	17 (73.9)	1 (2.4)
Patchy GGO	1 (4.3)	/
Definite ILA		
Nondependent ground-glass opacity	/	25 (59.5)
Nondependent reticular abnormality	/	16 (38.1)
Diffuse centrilobular nodularity	/	2 (4.8)
Traction bronchiectasis	/	1 (2.4)
Honeycombing	/	2 (4.8)
Architectural distortion	/	1 (2.4)
ILA score	1.22 ± 0.52	2.93 ± 1.72

Data are presented as No. (%) or mean ± SD. GGO, ground-glass opacity.

### 3.2 Quantitative CT measurements

Quantitative CT measurements are presented in Table 1. Consistent with previous studies (14), COPD patients with ILA had lower measurements of LAA-950, but no significant differences were observed between the three groups. In the spatial distribution of emphysema, similar to previous study (15), in COPD patients without ILA, the upper lobes had a higher proportion of LAA-950 than the lower lobes, such phenomenon can also be observed in COPD patients with definite ILA, and the difference was more obvious in the latter group; The proportion of LAA-950 in the upper lobes and lower lobes was similar in the those with equivocal ILA. The measurements of Pi10, segmental AWT, and segmental WA% exhibited highest in the definite ILA group, whereas lowest in the no ILA group. In Pi10 and segmental WA% measurements, significant differences were only observed between COPD patients with no ILA and those with definite ILA (*p* = 0.030 and *p* < 0.001, respectively). COPD patients with no ILA had higher measurements of TLCCT than those with ILA (*p* < 0.001). Meanwhile, the measurements of segmental AWT were significantly different between COPD patients with no ILA and those with equivocal ILA (*p* = 0.031) and between COPD patients with no ILA and those with definite ILA (*p* < 0.001). The segmental LD and segmental LA results showed no differences among the three groups.

### 3.3 AWT measurements according to ILA status

Quantitative CT measurements of AWT (including Pi10, segmental AWT, segmental WA%) according to ILA status were analyzed (Table 3). The measures of Pi10 and segmental WA% in COPD patients with definite ILA were significantly higher than those with no ILA. In addition, the segmental AWT measures in COPD patients with equivocal and definite ILA were significantly higher compared with those with no ILA. These results were shown in both univariate analyses and multivariate analyses adjusted by age, sex, BMI, smoking intensity, classification of COPD GOLD, lung function, slice thickness and scanner type. In the adjusted analyses, compared to COPD patients with no ILA, the measurements of Pi10, segmental AWT and segmental WA% were higher in definite ILA group with differences of 0.225 mm (*p* = 0.012), 0.152 mm (*p* < 0.001), 4.8% (*p* < 0.001) respectively.

**Table 3:** AWT measurements of equivocal and definite ILA patients compared to patients without ILA.

Variables	Unadjusted analysis		Adjusted analysis	
	β ± SE	*P* value	β ± SE	*p* value
Pi10 (mm)				
Equivocal ILA	0.121 ± 0.048	0.257	0.05 ± 0.109	0.651
Definite ILA	0.249 ± 0.085	0.004	0.225 ± 0.088	0.012
Segmental AWT (mm)				
Equivocal ILA	0.107 ± 0.041	0.010	0.087 ± 0.043	0.042
Definite ILA	0.159 ± 0.033	<0.001	0.152 ± 0.034	<0.001
Segmental WA%				
Equivocal ILA	2.3 ± 1.5	0.137	2.0 ± 1.5	0.186
Definite ILA	4.8 ± 1.2	<0.001	4.8 ± 1.2	<0.001

Data are presented as No. (%) or mean ± SD. See Table 1 legend for expansion of abbreviations. Adjusted analyses are adjusted for age, sex, BMI, smoking intensity, COPD GOLD Stage and lung function (FEV1/FVC, FEV1 percent predicted, FVC percent predicted, MMEF75/25 percent predicted, slice thickness and scanner type).

**Figure 4:**
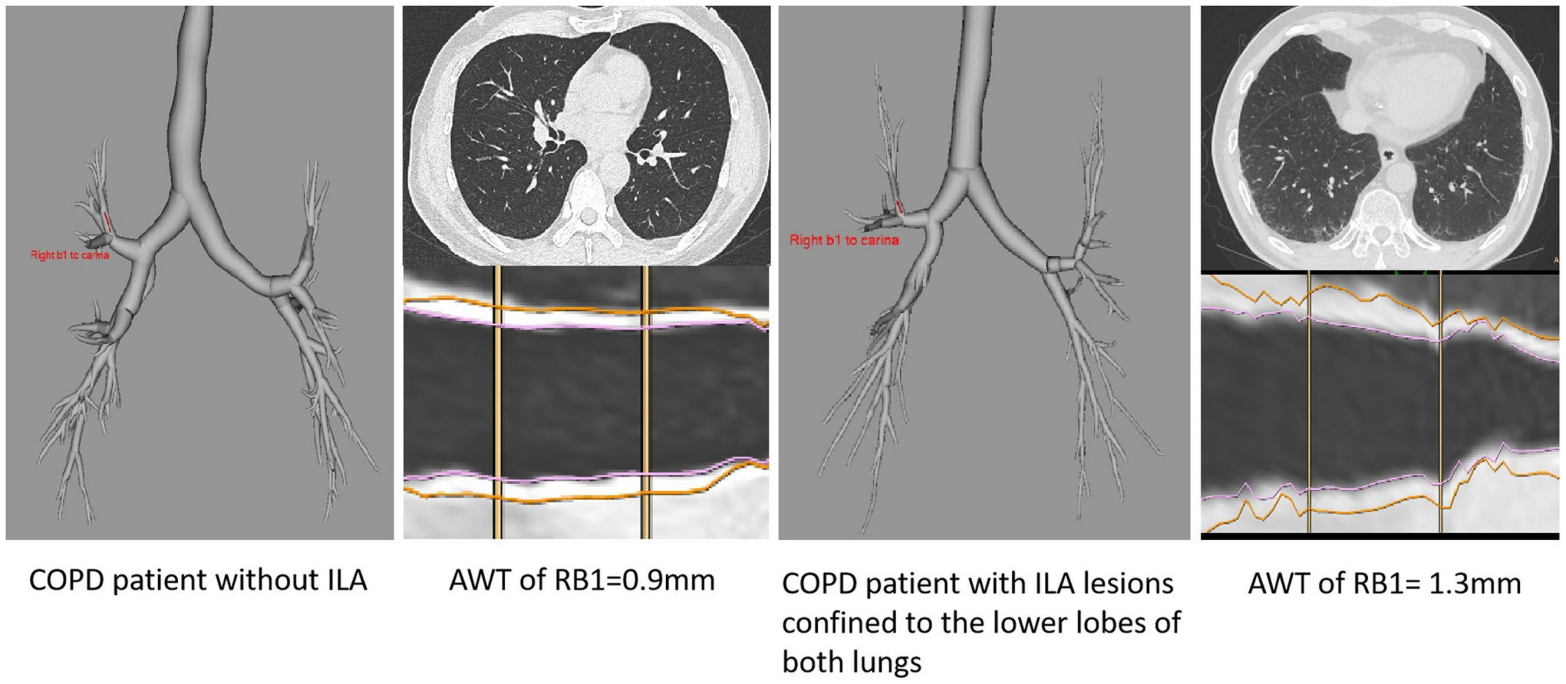
Entire airway tree slice of RB1 bronchi of COPD patients with and without ILA.

Furthermore, the findings of this association could be due to the ILA lesions only changing the adjacent airways, we excluded this possibility by selecting patients with ILA whose lesions were confined to the lower lobes of both lungs, and quantitative CT measurements of these patients (including AWT of RB1, RB4, LB1+2, the average of AWT of RB1, RB4, LB1+2, the average WA% of RB1, RB4, LB1+2) compared to patients with no ILA were analyzed (Table 4; Figure 4), We did not include Pi10 as the airway measure because it is a composite indicator. Similar higher measurements of AWT were noted in COPD patients whose ILA lesions were confined to the lower lobes, the results were observed in both univariate and multivariate analyses, which were adjusted for important covariates. The measures of AWT of RB1, the average AWT and WA% of these three segmental airways in COPD patients with ILA were significantly higher than those with no ILA. In addition, similar higher AWT measurements of RB4 and LB1+2 were also noted in COPD patients with ILA compared with those with no ILA, but no significant differences were observed between the two groups. In the adjusted analyses, compared to COPD patients without ILA, the average AWT and WA% measures of these three segmental airways were 0.189 mm (*p* = 0.001) and 3.897% (*p* = 0.013) higher in those with ILA, and higher AWT measures of RB1 with the difference of 0.287 mm (*p* < 0.001) in those with ILA.

**Table 4:** AWT measurements between patients whose ILA lesions were confined to the lower lobes of both lungs and patients without ILA.

Variables	Unadjusted analysis		Adjusted analysis	
	β ± SE	*P* value	β ± SE	*P* value
Segmental AWT*(mm)	0.152 ± 0.048	0.002	0.189 ± 0.053	0.001
Segmental WA%*	2.776 ± 1.333	0.040	3.897 ± 1.484	0.010
RB1 AWT (mm)	0.242 ± 0.064	<0.001	0.287 ± 0.068	<0.001
RB4 AWT (mm)	0.126 ± 0.084	0.138	0.178 ± 0.096	0.066
LB1+2 AWT (mm)	0.089 ± 0.057	0.120	0.101 ± 0.065	0.124

Data are presented as No. (%) or mean ± SD. See Table 1 legend for expansion of abbreviations. *Segmental AWT and segmental WA% were calculated as the average of three segmental airway (RB1, RB4, and LB1+2). Adjusted analyses are adjusted for age, sex, BMI, smoking intensity, COPD GOLD Stage and lung function (FEV1/FVC, FEV1 percent predicted, FVC percent predicted, MMEF75/25 percent predicted, slice thickness and scanner type).

## 4 Discussion

In this study, we demonstrate the quantitative measurements of AWT (including Pi10, segmental AWT, segmental WA%) are higher in COPD patients with equivocal ILA and definite ILA. And even in cases where ILA lesions were confined to the lower lobes of both lungs, thicker AWT were observed in the upper lobes of both lungs and in the right middle lobe of the lungs in COPD patients with ILA than those without ILA. But all of the lung function (including FEV1/FVC, FEV1% predicted, FVC% predicted, MMEF_75/25_% predicted) we enrolled showed no differences between these groups.

As far as we know, there have been limited studies employing quantitative CT to investigate AWT in COPD patients with ILA. Similar to our findings, Miller et al. (7) observed the higher Pi10 measurements in ILA and IPF patients in three separate centers (COPDGene, ECLIPSE, Framingham Heart Study). However, in a small sample study by Li et al. (8) (54 ILA patients vs. 18 healthy non-smokers), no difference in Pi10 measurements was observed between the two groups. In addition, as Pi10 and segmental WA% are comprehensive measurement index, their increases may be due to the increase of AWT or factors that may cause airway stenosis (such as mucus in airway cavity) (7). Therefore, in our study, the actual AWT, LD and LA of the five segments of airways were also measured. Even though the technical limitations made it impossible to measure the diameter of the airway less than 2 mm, numerous studies have demonstrated that larger airway wall thickness measurements can serve as a reliable surrogate for smaller airway measurements, and that the inflammatory response observed in smaller airways can also be detected in larger airways (16–18). Our study observed higher Pi10 and actual AWT measures in patients with ILA compared to those without ILA, while no significant differences in segmental LD and LA measures were observed between patients with equivocal/definite ILA and those without ILA, this ruled out the possibility those factors which result in narrowing of the airway lumen had effect on segmental WA% measures, nevertheless, the effect on Pi10 measurements cannot be excluded because the calculation of Pi10 also includes other airways besides the segmental airways. These findings suggest that airway wall thickening occurs in ILA patients, and given the genetic and genomic similarities between ILA and IPF, airway involvement may play a role in the pathogenesis of these diseases.

The data on the prevalence of ILA in asymptomatic smokers are increasing (mainly due to ongoing CT-based trials for lung cancer screening and other diagnostic purposes) (19, 20), in our study, the incidence of ILA was 41.4%, and definite ILA and equivocal ILA accounted for 26.8 and 14.6% respectively, which is higher than the proportion of 13.5–22.2% reported among smoking cohorts (21, 22), but similar to the prevalence of 48% in Framingham Heart studies and 43% in COPD Gene (23). As previous study have shown, increasing age and tobacco smoke exposure are clear risk factors for ILA (19, 24), we did found the COPD patients with definite ILA tended to be older, with each 1-year increase in age, the risk of definite ILA increased by 6.9%. Because of the close relationship between ILA and aging, the reticular abnormalities are sometimes considered to be part of the normal spectrum of senescent lung (25). However, based on the present studies, ILA is a risk factor for acute exacerbation and increased risk of mortality independent of age, highlighting its clinical importance (2, 26). Inconsistent with previous studies, the smoking intensity showed no difference among these groups in our study.

While the clinical significance of ILA is becoming more recognized, the etiology and natural history of ILA, ILD, and IPF remain unclear resulting in a lack of effective therapeutic techniques. Some studies have proposed the correlation between airway epithelial biology and IPF (4). Since ILA may have common pathogenesis with ILD and IPF (6), this study hypothesized that the airway may be involved in the pathogenesis of ILA, and evaluated the AWT of ILA patients from the perspective of imaging to verify this hypothesis. This provides additional support for the possible involvement of the airway in the pathogenesis of ILA and may improve understanding of the natural course of ILD and IPF. Considering previous studies have showed ILA was a risk factor for acute exacerbation and ultimately may affect COPD mortality, moreover, ILA was proved to be associated with an increased rate of dying from pulmonary fibrosis (2, 9, 23), ILA may represent a precursor of pulmonary fibrosis development in COPD patients.

Studies utilizing quantitative CT to evaluate the correlation between AWT and clinical course have primarily focused on COPD populations. Gietema et al. (16) reported that an increase in AWT was linked to a decrease in quality of life in patients with COPD, while Johannessen et al. (18) observed that an increase in AWT was linked to respiratory mortality in patients with severe emphysema. In addition, several studies have demonstrated that thicker AWT in COPD patients is associated with poorer lung function, higher risks of exacerbation of acute COPD, and higher scores on the St. George breathing questionnaire and body index (BMI, airflow obstruction, dyspnea, and exercise capacity) (27–29). All these studies provide evidence that airway wall thickening was associated with worse clinical outcomes.

Because ILA and COPD share similar risk factors (such as smoking, male gender, and old age), the two diseases often co-exist in patients, previous studies have reported a higher incidence of ILA in COPD patients than in smokers (22). However, few studies have investigated the relationship between ILA and COPD (19, 30). COPD is a heterogeneous disease, emphysema and airway disease can contribute independently to lung function decline (15). Considering the AWT measures increased in the ILA group, we speculated that COPD patients with ILA would exhibit poorer lung function than COPD patients without ILA. However, the statistical results were not consistent with our hypothesis, and all of the included lung function (FEV1/FVC, FEV1% predicted, FVC% predicted, MMEF75/25% predicted) were not different between the patients with equivocal/definite ILA and the patients without ILA. This result may be related to the fact that the majority of our patients with COPD were GOLD I stage and GOLD II stage (144/157, 91.7%). The relationship between ILA and spirometry results has also remained controversial in several studies evaluating the relationship between ILA and COPD (20). In some cohorts, the additional presence of ILA features was associated with a higher predicted percentage of FEV1 and a lower percent predicted diffusing capacity of the lung for carbon monoxide (DLCO) (9, 22, 31). similar to our study, some cohort studies showed there was no significant difference in FEV1 and FVC between patients with and without ILA (32), and in ECLIPSE cohort, significant higher FEV1 and FEV1/FVC were observed in COPD patients with ILA compared to no ILA (23). Interestingly, a recent study (33) based on smokers revealed the combination of ILA and emphysema may lead to a relative preservation of FVC, FEV1 and FEV1/FVC, but not in DLCO. Their findings demonstrated ILA can result in a relative preservation of measures of spirometry in patients with emphysema, that may lead clinicians to false assumptions about disease severity when spirometry is used alone, this study might further help us explain why the lung function (including FEV1% predicted, FVC% predicted) exhibited slightly higher in the COPD patients with definite ILA than those without ILA. Besides, note that restrictive and obstructive pulmonary diseases can have opposite effects on lung volume, which makes individual spirometry less sensitive in detecting clinically relevant changes, highlighting the role of CT in assessing this disease (5, 9), also Kahnert et al. (34) suggested Pi10 can be seen as an imaging biomarker for the course of COPD and response to treatment. In addition to lung function studies, previous studies have reported that ILA patients with COPD are associated with a higher risk of all-cause mortality, more respiratory symptoms, more severe clinical disease severity, reduced exercise capacity, and higher mortality (9, 20, 23, 35, 36).

There are several limitations to our study. Firstly, the sample size was small, and the number of ILA patients was relatively low. Additionally, it was a retrospective study conducted in a single center, which means that the evidence level was moderate. To address these limitations, future studies should be prospective, multi-center, and have a larger sample size. Second, because CT imaging was only performed in supine position, the high subpleural attenuation area in the lower lobe of both lungs caused by some pulmonary blood deposition effect was also classified into ILA population, which resulted in a slightly higher incidence of ILA patients in this study. Third, the generalization of the results is limited. The patients’ inspiratory status, different scanners, slice thickness and reconstruction kernel may affect the measurement results. In order to reduce these interference factors, we tried to guide the patients to maintain a stable full inspiratory position and made different scanners and slice thickness were distributed equally between groups. Fourth, the available lung function indicators in this study are not perfect, and lack of indicators that can reflect patients’ restrictive ventilation disorder and lung diffusion function (TLC% predicted, DLCO% predicted). Fifth, the proportion of males in the study population is quite high, and due to the physiological differences in bronchial wall thickness between males and females, this may limit the generalization of the study results. Sixth, in our study, the measurements of AWT and lung function were measured at only one point in time, therefore, future longitudinal assessments of AWT would be needed.

## 5 Conclusion

In conclusion, our study shows higher AWT measures in COPD patients with equivocal and definite ILA compared to patients without ILA. These findings suggest that the airway may be involved in the pathogenesis of ILA from an imaging perspective. However, our study did not observe a worse pulmonary function in COPD patients due to the prevalence of ILA.

## Data availability statement

The original contributions presented in the study are included in the article/Supplementary material, further inquiries can be directed to the corresponding author.

## Ethics statement

The studies involving humans were approved by the Ethics Commission of Wuhan Union Hospital. The studies were conducted in accordance with the local legislation and institutional requirements. Written informed consent for participation was not required from the participants or the participants’ legal guardians/next of kin in accordance with the national legislation and institutional requirements.

## Author contributions

YJ: Conceptualization, Data curation, Formal analysis, Methodology, Writing – original draft, Writing – review & editing. LC: Conceptualization, Data curation, Methodology, Writing – review & editing. JY: Data curation, Formal analysis, Investigation, Methodology, Software, Writing – review & editing. XY: Data curation, Formal analysis, Writing – review & editing. FY: Supervision, Validation, Visualization, Writing – review & editing.
