# Supplementary material for: Quantitative assessment of airway wall thickness in COPD patients with interstitial lung abnormalities
Source: Front Med (Lausanne). 2023 Dec 7;10:1280651. doi: 10.3389/fmed.2023.1280651 (PMC10749311; doi:10.3389/fmed.2023.1280651)
Supplement: Supplementary file 1 [file Table_1.DOCX]

Supplemental Table1 Evaluation of age for the risk of COPD patients with equivocal ILA and definite ILA.

| subgroups | Unadjusted analysis | | | Adjusted analysis | | |
| --- | --- | --- | --- | --- | --- | --- |
|  | OR | 95%CI | P value | OR | 95%CI | P value |
| Equivocal ILA | 1.035 | 0.982-1.091 | .202 | 1.046 | 0.989-1.105 | .115 |
| Definite ILA | 1.064 | 1.02-1.11 | .004 | 1.069 | 1.024-1.117 | .003 |

See Table 1 legend for expansion of abbreviations.

Adjusted analyses are adjusted by sex, BMI, smoking intensity, COPD GOLD Stage.
